# An Imported Case of Echinococcosis in a Pregnant Lady with Unusual Presentation

**DOI:** 10.1155/2013/753848

**Published:** 2013-01-17

**Authors:** Ahmed Al-Ani, Abdul-Naser Elzouki, Rashid Mazhar

**Affiliations:** ^1^Department of Medicine, Hamad General Hospital, Hamad Medical Corporation, P.O. Box, 3050, Doha, Qatar; ^2^Department of Surgery, Hamad General Hospital, Hamad Medical Corporation, P.O. Box 3050, Doha, Qatar

## Abstract

A 31-year-old Syrian pregnant (25-week duration) patient who was managed as a case of bronchial asthma for one year because of chronic cough and progressive breathlessness presented to the Accident and Emergency Department at Hamad General Hospital, Doha, with cough and shortness of breath. On the day of admission, she coughed large amount of yellowish watery material which was salty and sour in taste. She was found by radiological investigations to have multiple giant hydatid cysts (three in the lungs and one in the liver), with ruptured cyst in the left lung. We report here an unusual case of giant multiple pulmonary hydatidosis localized in the lungs and masquerading as bronchial asthma in a pregnant lady. The case represents an uncommon situation of a common disease of rupture of hydatid cyst of the lung in a pregnant lady in her 3rd trimester.

## 1. Introduction

Human echinococcosis was first described in ancient times by Hippocrates, as “cysts full of water” in a human liver [1, 2]. Al-Rahzes, the famous physician, subsequently wrote on hydatid cyst of the liver about thousand years ago [3]. Currently, hydatid disease is classified among the most neglected parasitic diseases [4, 5]. Infection with *Echinococcus granulosus* is the most common form of echinococcal infection in humans. The parasite involves dogs (the definitive hosts) and sheep (the intermediate hosts), and in the definitive canine host, the adult worm resides in the intestine, and its eggs, or oncospheres, are released into the feces [6]. Because humans play the same role of intermediate hosts in the tape warm life cycle as sheep, humans also become infected by ingesting tape warm eggs from an infected carnivore [7]. This occurs most frequently when individuals handle or contact infected dogs or other infected carnivores or inadvertently ingest food or drink contaminated with fecal material containing tape worm eggs [8]. The eggs can also be inhaled, causing primary lung disease [9]. When an oncosphere is ingested by a suitable intermediate host, it penetrates the intestinal wall and migrates by way of the portal circulation to the liver, where most oncospheres are deposited. However, some of the organisms are not filtered by the liver and are trapped in the pulmonary circulation [10]. 

An alternative path to the lung is the intestinal lymphatics, with entry into the circulation by way of the thoracic duct. A small proportion of oncospheres may be disseminated systemically and may infect any organ, including the central nervous system, myocardium, and bone. Within the chest, echinococcosis can primarily involve the pleural cavity [10], mediastinum [6], and chest wall. Pulmonary echinococcosis can follow intrathoracic rupture of a cyst of the liver [11].

Once an oncosphere is deposited in tissue, the metacestode, or hydatid cyst, develops [8]. The metacestode (echinococcal cyst) is a fluid-filled, spherical, unilocular cyst. Each cyst is surrounded by a host-produced layer of granulomatous adventitial reaction. Small vesicles called brood capsules bud internally from the germinal layer and produce multiple protoscolices by asexual division. In humans, the slowly growing hydatid cysts can attain a volume of several liters and contain many thousands of protoscolices [12]. Despite some progress in the control of echinococcosis, this zoonosis continues to be a major public health problem in several countries, and in several others it constitutes an emerging and reemerging disease.

## 2. Case Report

This 31-year-old pregnant (25-week duration) Syrian lady was admitted to the female medical ward through the Accident and Emergency Department at Hamad General Hospital, Doha, with two-days history of progressive shortness of breath and dry cough. Her history dated back to one year, when she started to complain of dry cough and shortness of breath. She was treated with bronchodilators at medical outpatient clinics as a case of allergic bronchitis with slight improvement in her symptoms. One month before this admission, the patient was referred from women hospital to chest clinic because of progressive shortness of breath where she was clinically diagnosed as a case of bronchial asthma in a pregnant lady based on history and clinical examination (no chest X-ray or pulmonary function tests were performed at that time). She was started again on two bronchodilator inhalers (i.e., Pulmicort Turbuhaler and Ventolin Inhaler). Despite taking these inhalers, the patient continued to have shortness of breath on mild exertion. On the day of admission, she coughed large amount (around 100 ml) of yellowish watery material which was salty and sour in taste. There was no history of haemoptysis, fever, night sweating, wheeze, itching, or weight loss. There was no history suggestive of chest trauma. Her obstetric medical history was relevant for a history of abortion one year ago, and at the time of admission she was pregnant with 25-week duration. She was not a smoker and had no previous contact with sheep, dogs, or birds or contact with sick persons. She was born and lived in Damascus, Syria, and recently moved to Doha, Qatar, with her husband. 

Physical examination revealed pale but not jaundiced lady. Vital signs revealed blood pressure 110/70 mmHg, pulse 130 beats per minute, temperature 36°C, respiratory rate 22 per minute, and O_2_ saturation 96% at room air. There was no cyanosis or lymphadenopathy. 

Chest examination revealed dull percussion note at both lung bases and decreased breath sounds in left lower zone without ronchi or crepitations. Abdominal examination was remarkable for palpable uterus of around 25-week duration. The cardiovascular and neurological examination was unremarkable. 

Laboratory investigations revealed total leucocytes count of 9.3 cells/cumm, neutrophils of 80%, eosinophil of 0%, haemoglobin of 10.7% gm, MCV of 92, and platelets of 315. ESR was 67 mm/hr and C-reactive protein was of 83 mg/L (normal: <5). All routine blood chemistry including renal tests and liver enzymes was within normal ranges. Bleeding and clotting times were normal, serum immunoglobulin E level was 778 KU/L (normal = 0–114), HIV: nonreactive, HBsAg: negative, and anti-HCV: negative. Electrocardiogram showed normal sinus rhythm. 

**Figure 1:**
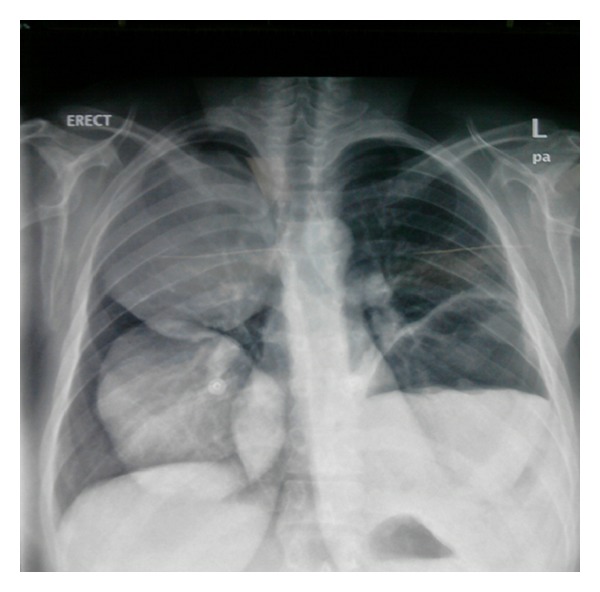
Chest X-ray revealed two fairly large unruptured hydatid cysts in the right lung and ruptured hydatid cyst in the left lung.

**Figure 2:**
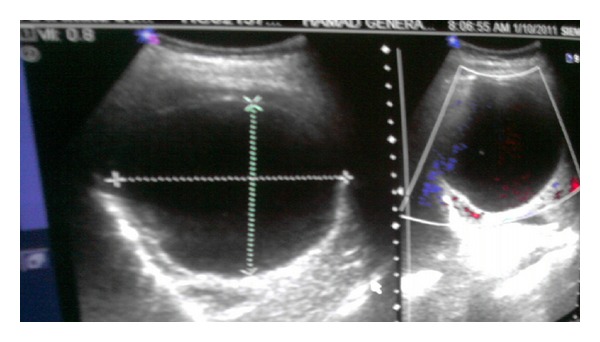
Abdominal ultrasound revealed a well-defined cystic lesion in the right liver lobe, likely hydatid cyst.

**Figure 3:**
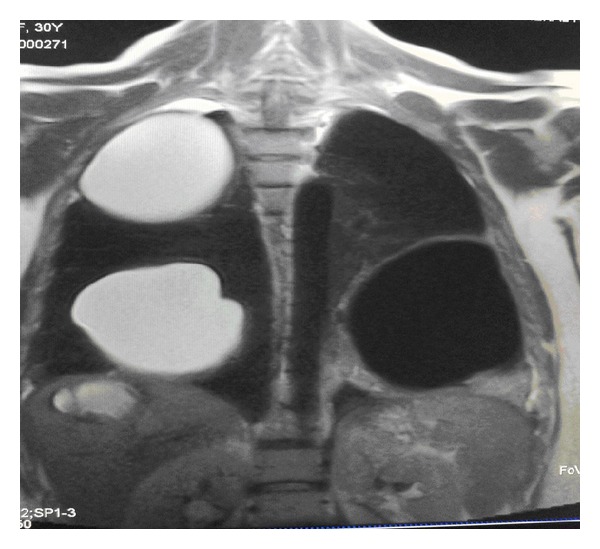
MRI of the thorax showed ruptured hydatid cyst of the left lung and multiple other intact hydatid cysts of the right lung and liver.

Chest X-Ray revealed two well-defined large cystic lesions involving the right upper, mid, and lower lung zones and another well-defined cystic lesion with central opacity with air fluid level involving the left lower lung zone with obliteration of left costophrenic angle. Findings fit with multiple giant pulmonary hydatid disease with ruptured cyst with air fluid level or loculated pleural effusion in the left side (Figure 1). Abdominal ultrasound showed a well-defined anechoic cystic lesion in the right liver lobe with internal echoes, measuring 9.3 × 8.7 cm, impressive of hydatid cyst (Figure 2). The MRI of the thorax findings is shown in Figure 3. Pelvic ultrasound showed single viable fetus with cephalic presentation and normal amount of amniotic fluid volume. 

Her clinical presentation was most likely due to complete evacuation of left lung hydatid cyst, which was partially ruptured at the day of admission.

Albendazole treatment was discussed with the patient but she refused to use it because of the potential risk to the fetus.

 The patient was managed by multidisciplinary team and cesarean section operation was performed under spinal anesthesia at 34 weeks of pregnancy. The patient gave birth to a normal healthy baby, the operation was uneventful, and the histopathology of the placenta showed no significant pathology. 

**Figure 4:**
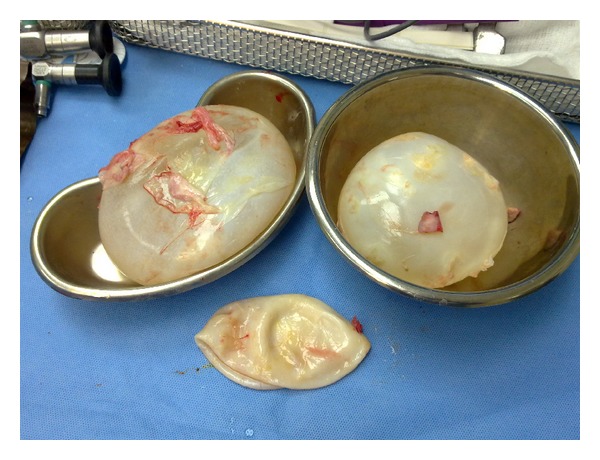
Postoperative removed three hydatid cysts.

The patient was followed in medical outpatient, and 6 weeks after delivery she was started on oral albendazole 15 mg/kg/day for two weeks then she was admitted again to hospital and single lung anesthesia right thoracotomy, pulmonary cystotomy, and transdiaphragmatic hepatic cystotomy were done uneventfully. Two cysts were removed from the right lung, larger cyst size was 12 cm × 11 cm × 3 cm, and the smaller cyst size was 10 cm × 10 cm × 1.5 cm, as well as one cyst removed from liver of 13 cm × 12 cm × 3.5 cm size (Figure 4). Histopathology of these cysts was consistent with hydatid disease. Patient was discharged on the 7th day after surgery and was kept for one month on albendazol as a postoperative prophylactic measure, and another surgery to remove the ruptured left lung cyst was done 8 weeks later with smooth postoperative course. 

## 3. Discussion

 Most primary infections of hydatid disease consist of a single cyst; however, 20%–40% of individuals have multiple cysts or multiple organ involvement [13], the liver being the most common site (>65%) followed by the lung (25%) and less frequently in other organs. Furthermore, hepatic cysts are found in approximately 10% to 25% of cases of pulmonary hydatid [9]. Moreover, pulmonary hydatid disease affects the right lung in approximately 60% of cases, 30% exhibit multiple pulmonary cysts, 20% bilateral cysts, and 60% are located in the lower lobes [14, 15]. The patient has multiple organ involvement (i.e., lungs and liver) and three cysts involved her both lungs, two cysts in the right lung, and one cyst in the left lung. 

The prevalence rate of echinococcal disease is high in the Middle East, particularly in Iraq, Syria, and Saudi Arabia [16–18].

In Qatar, echinococcal disease is seen mainly in people coming from endemic areas and the disease is rare in Qataris (in unpublished data 27 cases of echinococcal disease diagnosed in the last 12 years only four patients are Qatari).

 The patient was living since birth in Syria and came to Qatar one year before hydatidosis was diagnosed; however, the large size of the cysts suggests that she has got the infection decades earlier, when the patient resided in Syria. 

Despite a plethora of publications on echinococcosis, little is known about the disease in pregnant women. The incidence of the disease in pregnancy has been found as low as 1 in 20,000–30,000 [4], and usually it is asymptomatic but it was reported to cause anaphylaxis when ruptured intra-abdominally [19] and obstructed labour [20]. Most intact lung cysts are discovered incidentally on chest X-ray which probably was delayed in our patient because she was pregnant. Modalities that do not use ionizing radiation, such as ultrasonography and magnetic resonance imaging, should be the preferred examinations for evaluating an acute condition in a pregnant patient. However, no examination should be withheld when an important clinical diagnosis is under consideration. Exposure to ionizing radiation may be unavoidable, but there is no evidence to suggest that the risk to the fetus after a single imaging study is significant [21]. 

Occasionally, an unruptured cyst results in cough, haemoptysis, or chest pain [22]. Subsequent clinical features of *Echinococcal granulosus* infection depend upon the cyst site and size. Small cysts may remain asymptomatic indefinitely, but cysts may enlarge to more than 20 cm in diameter and cause symptoms by compressing adjacent structures. However, symptomatic hydatid disease of the lung more often follows rupture of the cyst.

The cyst may rupture spontaneously or as a result of trauma or secondary infection [23]. Our patient was complaining of chronic cough and shortness of breath on exertion and was labeled as long-term asthma. 

Her symptoms increased with progression of pregnancy and she presented to the emergency department with cough because of ruptured pulmonary cyst. Her chest X-ray showed three pulmonary cysts, one of them was already ruptured. Treatment of hydatid disease during pregnancy is challenging for fear of rupture, anaphylaxis, preterm labour, and intrauterine growth restriction due to large hepatomegaly. 

Albendazole is among the category C of drugs approved for use in pregnancy. The physician may administer the drug if the benefit for the patient outweighs the potential harm to the fetus. Albendazole may be more safely administered if the organogenesis of the fetus is completed [24]. 

No clear-cut guidelines are available on management on account of paucity of reported cases and, to the best of our knowledge, till now there is no consensus of management of hydatid disease during pregnancy. 

In view of the progressive dyspnoea that the patient had and because of the huge size of the unruptured pulmonary and liver cysts, the gynecologist decided to deliver the patient by cesarean section at 34 weeks of pregnancy.

After delivery, patient was managed surgically to remove the right pulmonary cysts in the first operation and subsequently the left pulmonary cyst was resected after 8 weeks. After delivery, she was given albendazole for a total of three-months period. 

We presented a case of multiple pulmonary hydatid disease in a pregnant lady missed for a substantial period of time and treated as a case of bronchial asthma because chest X-ray was not done in a patient with chronic cough. Physicians treating patients from endemic areas or working in endemic areas should be aware of hydatid disease and that it can endanger the life of the mother and the baby if untreated. There should be an increase in public awareness of the global nature of the disease. Moreover, we reported an uncommon situation of a common disease of ruptured hydatid cyst of the lung in a pregnant lady in her 3rd trimester. The primary choice is surgical management of pulmonary and hepatic cysts simultaneously through the thoracic route, is convenient, and should not be delayed. Albendazole might help with surgical removal of the cyst. The ultimate aim remains, however, to have proper control measures and prevent these cestode infestations.
